# A Short Empathy Paradigm to Assess Empathic Deficits in Schizophrenia

**DOI:** 10.3390/bs10020041

**Published:** 2020-01-24

**Authors:** Foteini Peveretou, Sina Radke, Birgit Derntl, Ute Habel

**Affiliations:** 1 Department of Psychiatry, Psychotherapy and Psychosomatics, Faculty of Medicine, RWTH Aachen University, Pauwelsstrasse 30, 52074 Aachen, Germany; sradke@uos.de (S.R.); uhabel@ukaachen.de (U.H.); 2 Jülich Aachen Research Alliance—BRAIN Institute I: Brain Structure–Function Relationships: Decoding the Human Brain at Systemic Levels, Research Center Jülich GmbH and RWTH Aachen University, Wilhelm-Johnen-Strasse, 52425 Jülich, Germany; 3 Department of Psychiatry and Psychotherapy, Medical School, University of Tübingen, Calwerstrasse 14, 72076 Tübingen, Germany; Birgit.Derntl@med.uni-tuebingen.de

**Keywords:** affective responsiveness, emotion recognition, empathy, perspective taking, psychopathology, schizophrenia

## Abstract

Empathy is important for successful social interaction and maintaining relationships. Several studies detected impairments in empathic abilities in schizophrenia, with some even indicating a broader deficit in several components, including emotion recognition, perspective taking, and affective responsiveness. The aim of our study was to validate a short version of the previous empathy paradigm as a reliable and easily applicable method to assess empathic deficits in patients with schizophrenia potentially within clinical routine. To do so, we applied the short version to 30 patients (14 females) diagnosed with schizophrenia meeting the DSM-5 criteria and 30 well-matched healthy controls (14 females). The data analysis indicates a significant empathic deficit in patients due to worse performance in all three domains. We managed to replicate most of the findings of our previous study. In contrary to the previous study, significant correlations between performance in the empathy tasks and psychopathology occurred: the severity of negative symptoms was negatively associated with performance in the emotion recognition task and the affective responsiveness task. Gender did not significantly affect performance in the empathy tasks. Regarding the results, our short empathy paradigm appears to be a valid method in assessing empathic impairments in schizophrenia that may be useful in clinical routine.

## 1. Introduction

Empathy is defined as the ability to share and understand the emotional states of others and respond to them appropriately. It plays an important role in maintaining successful social relationships [1]. Empathy enables individuals to share experiences, feelings, and needs and provides an emotional connection that fosters pro-social behavior [2]. Social dysfunctions characterize many psychiatric disorders and can be induced or intensified by deficits in empathy and the Theory of Mind (ToM) [3,4,5]. ToM refers to the ability to infer our own and other person’s mental states [6]. It is also described as mentalizing and part of cognitive empathy.

Three main empathy components were described by Decety and Jackson (2004): (a) perception of emotions in oneself and others, (b) the ability to put oneself in the place of others, taking their own perspective and infer their feelings (perspective taking), and (c) the ability to share and experience the feelings of others (affective responsiveness).

Furthermore, studies suggest that deficits in social cognition might be a marker for psychiatric disorders, including schizophrenia and autism [7,8]. In schizophrenia, deficits in social cognition and metacognition lead to poor social functioning and isolation [9]. Plenty of studies so far demonstrated a clear empathic deficit in all empathic domains in schizophrenia [10,11,12,13,14,15,16]. In these studies, empathic deficits were assessed with different methods, ranging from computer tasks to self-report questionnaires, all detecting significant empathic impairments in patients.

Concerning the evaluation of several components of empathy, Derntl and colleagues (2009) used three different tasks, one for every empathic component, as well as self-report questionnaires. The authors revealed deficits in all core empathic components in schizophrenia and thus an overall impairment in empathy in comparison to well-matched healthy controls.

The aim of the present study is to validate a short version of the same empathy paradigm that has been used in our previous study [10] as a reliable method to assess empathic deficits in schizophrenia patients. Based on [10], we expected a significant empathic impairment in patients with schizophrenia, no significant differences in reaction times between the two groups, and no effect of gender on empathic performance. Regarding emotion-specific performance, we expected highest accuracy for happy stimuli in all empathy tasks. Moreover, we were specifically interested whether psychopathological parameters, such as the duration of illness and symptom severity, affect empathic abilities. Based on a previous meta-analysis, we further expected significant correlations between the performance in the empathy tasks and the self-report-empathy measurements [16].

## 2. Materials and Methods

### 2.1. Sample

Thirty patients fulfilling the diagnostic criteria for schizophrenia (DSM-5) and 30 healthy controls (HC) matched for gender, age, and education (school years) participated in this study. The exclusion criteria for all participants were substance abuse in the past two months, any mental disorders other than the targeted psychiatric disorder, and impaired intelligence. All participants were fluent in German. In patients, the severity of the symptoms was assessed with the Positive and Negative Syndrome Scale (PANSS) [17]. Patients with schizophrenia also completed the Beck Depression Inventory (BDI-2, [18]) to assess mood symptoms.

All subjects gave written informed consent after a complete study description. The study was approved by the local ethics committee (RWTH University Aachen, Approval number: EK 073/15).

Patients with schizophrenia (SZP) were recruited from the in- and out-patient units of the Department of Psychiatry, Psychotherapy and Psychosomatics, Medical School, RWTH Aachen University, Germany. All patients received atypical antipsychotic medication except for one patient who was treated with a typical antipsychotic agent. One female patient suffered from an alcohol addiction in the past and had been abstinent for 15 years, which is why we included her in our study. In three male patients, substance abuse was described but they had been clean for the past 2 months. Demographic and clinical characteristics are shown in Table 1.

### 2.2. Tasks

#### 2.2.1. Emotion Recognition

**Figure 1:**
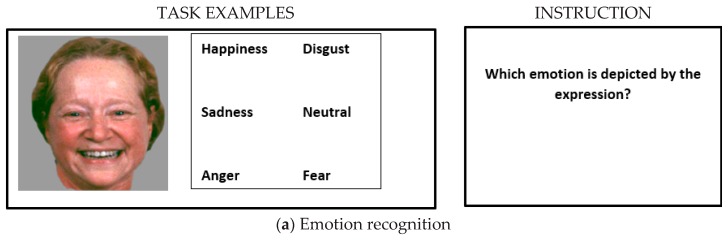

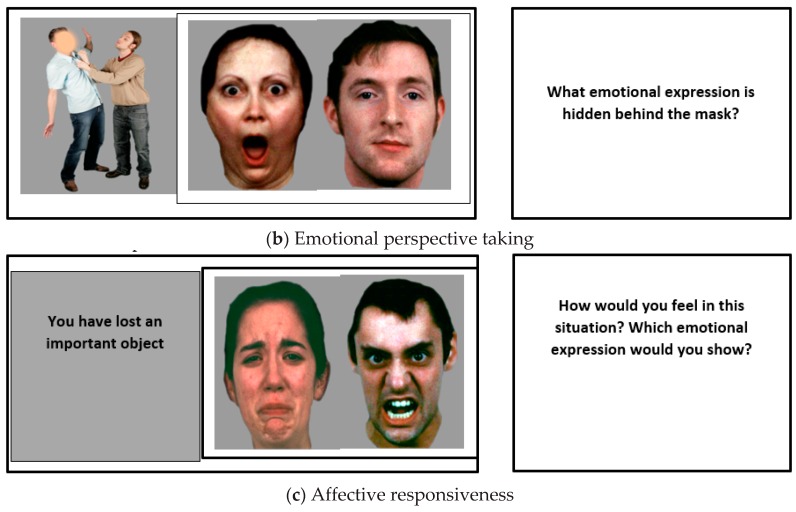
Illustration of the three tasks, measuring (**a**) emotion recognition, (**b**) emotional perspective taking, and (**c**) affective responsiveness as core components of the empathic ability. Task instructions are listed next to the example stimuli. Image adapted from [10].

Thirty-six photographs of Caucasian faces depicting five basic emotions (happiness, disgust, fear, anger, and sadness) and neutral expressions were presented on the left side of the screen. Facial stimuli were selected from a standardized stimulus set [19] that has been used repeatedly before [10,11,20,21,22]. On the right side of the screen, six verbal labels were presented, and participants were instructed to choose the correct emotion for every face (please see Figure 1). Responses were made by pressing the up/down arrow keys on the keyboard. The task was self-paced, i.e., without a time limit.

#### 2.2.2. Emotional Perspective Taking

Participants viewed 36 items that were each presented for 4s. The scenes showed two Caucasians involved in a social interaction thereby portraying the emotions described above and neutral scenes. The face of one person was masked and participants were asked to infer the corresponding emotional expression of the covered face. Responses were made via button press by selecting between two different emotional facial expressions or a neutral expression presented for 4 s after each scene. Facial alternatives were taken from the same pool of stimuli described above. One option was correct and the other was selected at random from all other choices.

#### 2.2.3. Affective Responsiveness

We presented 36 short written sentences describing real-life situations, which are likely to induce one of the basic emotions described above. Participants were asked to imagine how they would feel if they were experiencing those situations. Sentences were presented for 4s and the response format was the same as for emotional perspective taking.

Please see Figure 1a–c for examples of each task.

#### 2.2.4. Empathy Questionnaire

We administered one self-report questionnaire assessing cognitive and affective empathy: the German version of the Interpersonal Reactivity Index (IRI, [23]), the ‘Saarbrücker Persönlichkeitsfragebogen, SPF (Paulus, 2006). The SPF includes 16 items with four subscales, each made up of four items.

These subscales are taken directly from Davis (1983): (i) Perspective Taking: the tendency to spontaneously adopt the psychological point of view of others, (ii) Fantasy: taps respondents’ tendencies to transpose themselves imaginatively into the feelings and actions of fictitious characters in books, movies, and plays, (iii) Empathic Concern: assesses “other-oriented” feelings of sympathy and concern for unfortunate others, and (iv) Personal Distress: measures “self-oriented” feelings of personal anxiety and unease in tense interpersonal settings.

### 2.3. Statistical Analyses

For each task, performance was determined by the percentage of correct responses. To account for non-normality of the data, the percentage of correct responses was analyzed using Generalized Estimating Equations (GEE) with Emotion (six levels: happiness, sadness, anger, fear, disgust, neutral) as a within-subject factor and Group (SZP, HC) and Gender (male, female) as between-subject factors. In case of singularity of the Hessian Matrix, emotion categories, without variance within a group (fear for affective responsiveness), were excluded in order to satisfy convergence criteria. Statistical parameters are reported for type III model effects and follow-up pairwise comparisons of significant effects, which have been Bonferroni-corrected.

In the emotion recognition task, three patients and one control participant showed no correct responses for all trials of one facial emotion and were therefore excluded from the ANOVAs for this task. The same applied for 2 patients in the perspective taking task. For each task, the mean reaction times (RTs) of correct responses were calculated per emotion and subjected to a mixed-model ANOVA with Emotion (six levels) as a within-subject factor and Group (SZP, HC) and Gender (male, female) as between-subject factors.

Differences in self-reported empathy (SPF) were assessed using a mixed-model ANOVA for the four subscales (fantasy, empathic concern, perspective taking, distress, neutral) with Group (SZP, HC) and Gender (male, female) as between subject factors. Greenhouse Geisser corrected *p*-values are reported in cases of sphericity violation, and partial eta squares are listed as an indication of effect size. Post-hoc comparisons of the ANOVAs are Bonferroni-corrected. Mean accuracy values across the emotional categories for each of the three tasks were correlated with each other for patients and controls separately. Moreover, in patients, clinical parameters were correlated with behavioral performance (accuracy). The correlations were corrected for multiple comparisons.

## 3. Results

### 3.1. Emotion Recognition: Accuracy

The GEE analysis for percent correct revealed a significant main effect of Emotion, Wald-χ2(5) = 180.32, *p* < 0.001 (see Table 2 for means and follow-up comparisons), and a significant main effect of Group, Wald-χ2(1) = 4.23, *p* = 0.039. No other effects or interactions were significant, all Wald-χ2 < 9.83, *p* > 0.08. The main effect of Group was due to better performance in HC (*M* = 78.7%, *SD* = 7.7) than SZP (*M* = 74.3%, *SD* = 14.3).

#### 3.1.1. Emotion Recognition: Speed

The Emotion x Group x Gender ANOVA on the RTs showed a significant main effect of Emotion, *F*(5260) = 4.17, *p* = 0.005, partial *η*^2^ = 0.07 (see Table 3 for means and follow-up comparisons). There was also a significant main effect of Group, *F*(1,52) = 10.95, *p* = *0*.002, partial *η*^2^ = 0.17, which was due to longer RTs in SZP (*M* = 6788.3ms, *SD* = 3173.3) than HC (*M* = 4482.91ms, *SD* = 1711.36). All other effects were not significant, all *Fs* < 1.61, *ps* > *0*.18.

#### 3.1.2. Perspective Taking: Accuracy

The GEE analysis for percent correct revealed a significant main effect of Emotion, Wald-χ2(5) = 57.44, *p* < 0.001 (see Table 2), and a significant main effect of Group, Wald-χ2(1) = 19.80, *p* < 0.001. Other effects were not significant, except for the interaction of Gender-by-Group being marginally significant, Wald-χ2(1) = 3.81, *p* = 0.051.

The main effect of Group was due to higher accuracy in HC (*M* = 84.0%, *SD* = 7.5) than SZP (*M* = 69.2%, *SD* = 27.2). Decomposing the trendwise Gender-by-Group interaction indicated that males performed better than females only in HC, Wald-χ2(1) = 4.74, *p* = 0.029, but not in SZP, Wald-χ2(1) = 0.77, *p* = 381. Moreover, male HC performed better than male SZP, Wald-χ2(1) = 11.16, *p* = 0.001, but female HC and female SZP did not differ significantly, Wald-χ2(1) = 3.39, *p* = 0.07.

#### 3.1.3. Perspective Taking: Speed

The Emotion x Group x Gender ANOVA on the RTs showed a significant main effect of Emotion, *F*(5270) = 11.12, *p* < 0.001, partial *η^2^* = 0.17 (see Table 3), and a significant main effect of Group, *F*(1,54) = 7.56, *p* = 0.008, partial *η*^2^ = 0.12, which was due to longer RTs in SZP (*M* = 1870.9ms, *SD* = 379.2) than in HC (*M* = 1613.9ms, *SD* = 312.1). There was a significant interaction of Emotion-by-Group, *F*(5270) = 2.84, *p* = 0.022, partial *η*^2^ = 0.05. No other effects were significant, all *Fs* < 2.65, *ps* > 0.11. Decomposing the interaction revealed that SZP showed significantly longer RTs especially to sad and happy situations than HC (see Table 3 for means).

#### 3.1.4. Affective Responsiveness: Accuracy

The GEE analysis for percent correct revealed significant main effects of Emotion, Wald-χ2(4) = 36.41, *p* < 0.001 (see Table 2) and of Group, Wald-χ2(1) = 42.17, *p* < 0.001. There was also a significant Emotion-by-Gender interaction, Wald-χ2(4) = 10.95, *p* = 0.027. Other effects were not significant, all Wald-χ2 < 7.42, *p* > 0.12. The main effect of Group reflected higher accuracy in HC (*M* = 92.2%, *SD* = 5.9) than in SZP (*M* = 77.8%, *SD* = 20.3). Decomposing the Emotion-by-Gender interaction indicated that, irrespective of group, males performed better than females for disgust, Wald-χ2(1) = 5.53, *p* = 0.019, whereas females performed better than males for happy emotions, Wald-χ2(1) = 4.30, *p* = 0.038.

#### 3.1.5. Affective Responsiveness: Speed

The Emotion x Group x Gender ANOVA on the RTs showed a significant main effect of Emotion, *F*(5280) = 11.03, *p* < 0.001, partial *η*^2^ = 0.16 (see Table 3 for means and follow-up tests), and a significant main effect of Group, *F*(1,56) = 11.23, *p* < 0.001, partial *η*^2^ = 0.17, which was due to longer RTs in SZP (*M* = 1767.8ms, *SD* = 432.0) than in HC (*M* = 1447.3ms, *SD* = 284.3). No other effects reached significance, all *Fs* < 0.78, *ps* > 0.543.

#### 3.1.6. Inter-Task Relations

In SZP, accuracy scores of affective responsiveness and emotion recognition were correlated, *r* = 0.551, *p* = 0.002, and trend-wise also scores of affective responsiveness and perspective taking, *r* = 0.391, *p* = 0.032 (not significant after correcting for multiple testing). In HC, there were no analogous correlations (all *p*s > 0.52).

#### 3.1.7. Psychopathology and Performance

Emotion recognition accuracy was negatively correlated with the PANSS subscale negative symptoms (*r* = −0.617, *p* < 0.001). Accuracy in the perspective taking task was negatively correlated with PANSS general psychopathology (*r* = −0.501, *p* = 0.005). Affective responsiveness was negatively correlated with the PANSS subscales negative symptoms (*r* = −0.548, *p* = 0.002) and general psychopathology (*r* = −0.549, *p* = 0.002).

#### 3.1.8. Self-Reported Empathy

The Scale × Group × Gender ANOVA on the SPF scores showed a significant main effect of Scale, *F*(3168) = 24.22, *p* < 0.001, partial *η^2^* = 0.30, and a significant main effect of Gender, *F*(1,56) = 12.81, *p* = 0.001, partial *η^2^* = 0.19, which was due to higher scores in females (*M* = 14.4, *SD* = 2.1) than males (*M* = 12.6, *SD* = 1.8). There was a significant Scale-by-Group interaction, *F*(3168) = 12.76, *p* < 0.001, partial *η^2^* = 0.19. Decomposing this interaction revealed that SZP showed significantly higher scores on the subscale Personal distress (*M* = 12.9, *SD* = 3.5) and significantly lower scores on the subscale Perspective taking (*M* = 13.6, *SD* = 3.4) than HC (*M* = 9.4, *SD* = 3.5, and *M* = 16.2, *SD* = 2.6, respectively). The main effect of Scale was predominantly due to differences between scores of the (reversely coded) Personal distress scale and the Empathic concern and Perspective taking scales and is therefore not discussed further.

The duration of illness was not significantly correlated with self-reported empathy (all *p*s > 0.11).

## 4. Discussion

Our study investigated empathic abilities in patients suffering from schizophrenia and compared those with well-matched healthy subjects. In a previous study of our group [10], deficits in all empathic components were observed in schizophrenia using three separate tasks, one for each of the three empathic components: emotion recognition, perspective taking, and affective responsiveness [24]. The aim of this study was to validate a short version of the empathy paradigm to assess empathic deficits in schizophrenia that could be used as an economic and easily applicable version for clinical routine and further studies in this field.

**Figure 2:**
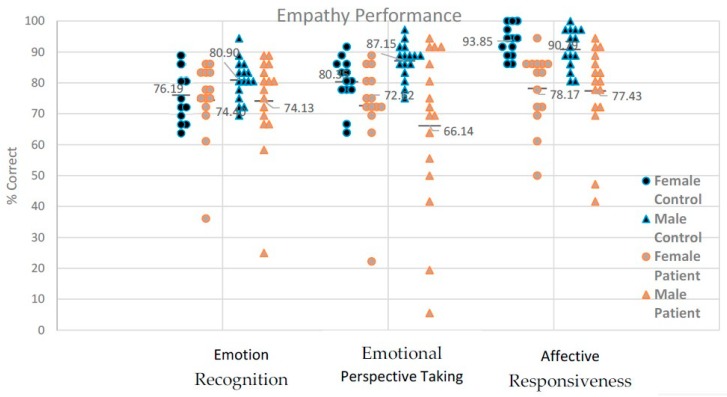
Performance (% correct and a line indicating mean group performance) in emotion recognition, emotional perspective taking, and affective responsiveness in schizophrenia patients and healthy controls.

As expected, patients with schizophrenia showed worse performance than healthy controls in all empathy components. This result supports the idea of an encompassing deficit in schizophrenia across the three empathic components. In contrast to our previous study [10], here, performance in the empathy tasks was correlated with symptom severity based on the PANSS scores. Negative symptom severity was associated with reduced accuracy in emotion recognition and affective responsiveness, while severe mixed symptoms were associated with reduced performance in perspective taking and affective responsiveness. Like in the previous study, self-reported empathy scores were not related to performance-based empathic abilities (See Figure 2).

Generally, the results replicate the findings of the previous study of our group except for the influence of psychopathology in empathic abilities in schizophrenia and response times. Therefore, our short empathy paradigm appears to be a valid method for assessing empathic impairments in patients with schizophrenia. It can be used in future studies in this field and may be more applicable and tolerable for patients since it is shorter (test duration is 20 min) and less taxing on patients.

### 4.1. Empathy Tasks

Ιn all three empathy tasks, patients with schizophrenia showed significant impairments compared to healthy controls, a result that comes to an agreement with the previous findings from Derntl et al. (2009). Over the past years there has been a considerable amount of literature that reported impairments in emotion recognition in schizophrenia and analyzed their impact on social functioning [12,25,26,27,28,29]. Although the origin of the observed empathic deficits remains unclear, many studies support the idea that the deficit constitutes a trait-like-nature in schizophrenia, through its stability over time, notwithstanding stage of illness [30,31,32,33] and its early appearance. This supports the idea that empathic dysfunctions represent a vulnerability marker in schizophrenia [10,11,21].

Similar to emotion recognition, the majority of the studies about perspective taking and affective responsiveness report significant deficits in schizophrenia in comparison to healthy controls [10,11,34,35,36,37].

Deficits in perspective taking appear in other studies to be a trait marker of the disorder. Schiffman et al. [38] investigated the perspective taking capacity among children that later did or did not develop schizophrenia spectrum disorders indicating clear impairment in those that suffered from the disease later. The participants were high-risk children, i.e., with at least one parent with a schizophrenia diagnosis, and control children, i.e., children with at least one parent with a psychiatric disorder except from schizophrenia or with no psychiatric disease. The results suggest that this facet of Theory of Mind (ToM) is impaired prior to disease manifestation.

Regarding affective responsiveness, healthy subjects again outperformed patients. In comparison to the previous study, gender differences responding to different emotions occurred with men showing higher accuracy for disgust scenes, while women outperformed men in the happy condition.

### 4.2. Reaction Times and Emotions

Contrary to our previous study [10], patients with schizophrenia responded significantly slower than healthy controls in all empathy tasks and for all emotions and neutral expressions. Patients with schizophrenia generally show a slower processing on most types of empathy tasks, such as facial and prosodic emotion recognition [39,40].

Regarding emotions, happiness was recognized the fastest and best for both groups in all empathy tasks, a result that corroborates the findings of Derntl et al. [10]. Happiness is the easiest emotion to recognize not only for healthy participants [41] but also for patients with schizophrenia [10,11,40,42,43,44].

### 4.3. Influence of Illness Characteristics

As hypothesized, significant influences of psychopathology on the performance in the empathy tasks occurred: For emotion recognition, greater severity of negative symptoms indicated worse performance. In perspective taking, performance was negatively correlated with general psychopathology. Moreover, mixed and negative symptom severity was related to poorer performance in affective responsiveness. Like in the previous study, there was no significant correlation between performance in the empathy tasks and the duration of illness.

Our results suggest that, in schizophrenia, mainly negative and mixed symptoms are associated with impaired abilities in all empathic components. Since most items in the Negative PANSS subscale mirror affective dysfunctions in schizophrenia, our results suggest that patients with severe negative symptoms such as blunted affect, passive social and emotional withdrawal, and poor affective rapport have larger deficits in processing the emotional state of another person but also their own. Several studies have found associations between overall empathy and negative symptoms in patients with schizophrenia [45,46,47,48,49].

Although most studies so far relate negative symptoms with greater impairment in processing the emotional states of others in schizophrenia, some demonstrate a significant correlation between the severity of positive symptoms and the performance in empathy tasks [25,50,51,52]. Moreover, other studies could not even detect any significant correlations between psychopathology and emotion recognition in schizophrenia [15,53,54,55,56,57]. The inconsistent findings show the difficulty to assess the exact influence of psychopathology on the different empathic components in schizophrenia. More studies with a larger number of participants are needed in order to understand the exact role of psychopathology for the empathic abilities in schizophrenia. Our findings, as well as the findings of previous studies, suggest that different aspects of empathy, such as cognitive and affective components or emotion processing of others and our own, are influenced differently by the different subtypes of schizophrenia based on the severity of specific symptoms. Other aspects, such as the heterogeneity of clinical characteristics with regard to symptom severity, psychosocial functioning, comorbidity, and chronicity, as well as the variety of tools that have been used in assessing symptom severity in schizophrenia, should also be considered.

### 4.4. Empathy Questionnaires and Gender

Like in the previous study, we also did not find significant correlations of the performance in the empathy tasks with self-report empathy based on the self-report questionnaire.

In comparison to the previous study, gender was not related with the performance in the empathy tasks. However, with regards to self-reported empathy, females reported generally higher scores than males across the whole sample. Plenty of studies support that in general, women outperform men in empathic abilities based on self-report-questionnaires and facial indexes of emotion [1,58,59]. In most studies so far, gender differences did not occur in experimental tasks and physiological measures [60,61,62]. This divergence supports the evidence of gender stereotypes and might appear biased through specific social expectations and gender-role stereotypes that picture women as more caring and empathic than men [58,63,64].

The influence of gender on empathy in schizophrenia, however, remains imprecise due to inconsistent findings so far. Mote and Kring [65] included in their meta-analysis 38 studies on gender differences in facial emotion processing in schizophrenia. Significant results occurred only in eight of those studies and in six of those, women outperformed men across the schizophrenia spectrum.

### 4.5. Limitations

Our main limitation in this study is the small sample size and we recognize that it might pose a statistical disadvantage. Since our main goal was to validate the short paradigm from our previous study, a similar sample size was chosen. Using the same empathy paradigm introduces similar limitations as in the previous study [10]. The same forced-choice answering format was used and thus analyses of error patterns were not possible. Additionally, restricting the response alternatives to two possibilities in two empathy tasks seems to have decreased the overall difficulty of the tasks.

## 5. Conclusions

Our main results correspond to the majority of studies that described an impairment of empathy in schizophrenia. Patients with schizophrenia show deficits in all empathic components, assessing emotional states of other people based on their expressions, understanding another emotional state and point of view in social interactions, and processing their own emotional state in different situations. The development of methods to improve empathic abilities thus is mandatory in the therapeutic process for schizophrenia. A main symptom of the disease is social isolation. Hence, an improvement in core components of empathy might lead to better integration of patients with schizophrenia, which may also impact the general therapy outcome and quality of life [34,66]. Using a short version of Derntl’s empathy paradigm, we detected significant deficits in patients with schizophrenia in all core components of empathy. In contrast to our previous’s study, significant influences of psychopathology on the performance in the empathy tasks occurred as expected. Based on our aim to validate the short version of Derntl’s empathy paradigm, our results support it as a valid method to assess empathic deficits in schizophrenia.

## Figures and Tables

**Table 1:** Sociodemographic characteristics of schizophrenia patients and controls, showing no significant difference in age and education between the two groups.

	Patients (*n* = 30)	Controls (*n* = 30)	*p*-Values
Gender f:m	14:16	14:16	
Mean years of age	39.3	39.5	0.951
Duration of illness (yrs)	11.9 (Range: 2 months-37 yrs)		
Education (yrs)	10.9	11.6	0.327
PANSS positive score	12.56		
PANSS negative score	14.66		
PANSS general score	26.46		
PANSS global score	53.7		

**Table 2:** Task performance across the whole sample and for the two subgroups, F: female, M: male, S: sum (presented as Mean [SD] in percent correct).

	Whole Sample (*n* = 60)	HC (*n* = 30)			SZP (*n* = 30)		
**Emotion recognition**		F	M	S	F	M	S
Happiness	91.7	95.0	95.0	95.0	90.0	86.7	88.3
Sadness	60.0	60.0	60.0	60.0	65.5	51.7	56.7
Anger	85.0	85.0	90.0	86.7	80.0	85.0	82.8
Fear	76.7	80.0	71.7	75.0	75.0	78.3	74.4
Disgust	63.3	56.7	75.0	66.2	55.0	66.7	58.9
Neutral	88.3	86.7	95.0	91.7	85.0	83.3	84.4
**Perspective taking**							
Happiness	86.7	91.7	96.7	93.3	81.7	78.3	80.0
Sadness	80.0	90.0	86.7	88.3	78.3	65.0	71.7
Anger	66.7	63.3	80.0	71.7	61.7	61.7	61.7
Fear	75.0	75.0	81.7	88.3	76.7	66.7	71.7
Disgust	75.0	75.0	85.0	80.0	73.3	68.3	70.0
Neutral	81.7	88.3	93.3	91.7	68.3	73.3	71.7
**Affective responsiveness**							
Happiness	90.0	93.3	98.3	96.7	81.7	85.0	83.3
Sadness	78.3	93.3	85.0	90.0	68.3	68.3	68.3
Anger	86.7	93.3	95.0	95.0	81.7	76.7	78.3
Fear	90.0	100	88.3	93.3	88.3	83.3	85.0
Disgust	90.0	98.3	93.3	95.0	86.7	81.7	85.0
Neutral	75.0	83.3	85.0	83.3	63.3	68.3	66.1

Note: Across the whole sample: (a) for emotion recognition, accuracy for the following emotions differed significantly from each other: anger vs. disgust, anger vs. sadness, anger vs. fear, disgust vs. happiness, disgust vs. neutral, happiness vs. fear, happiness vs. sadness, neutral vs. fear, neutral vs. sadness, fear vs. sadness. (b) Significant differences in perspective taking across the whole sample were evident in the following comparisons: anger vs. happiness, anger vs. fear, anger vs. neutral, anger vs. sadness, disgust vs. happiness, happiness vs. fear, fear vs. neutral. (c) For affective responsiveness, the following comparisons yielded significant differences across the whole sample: disgust vs. sadness, happiness vs. sadness; and neutral significantly differed from all others except for sadness.

**Table 3:** Reaction times across the whole sample and for the two subgroups (presented as Mean [SD] in ms).

	Whole Sample	HC	SZP
Emotion recognition	(*n* = 56)	(*n* = 29)	(*n* = 27)
Happiness	4310	3243	5376
Sadness	5942	5207	6676
Anger	5316	4692	5940
Fear	7014	5666	8361
Disgust	5816	3886	7745
Neutral	5419	4204	6633
Perspective taking	(*n* = 58)	(*n* = 30)	(*n* = 28)
Happiness	1531	1344	1718
Sadness	1745	1532	1957
Anger	1891	1761	2021
Fear	1825	1787	1863
Disgust	1741	1667	1814
Neutral	1691	1589	1792
Affective responsiveness	(*n* = 60)	(*n* = 30)	(*n* = 30)
Happiness	1450	1291	1609
Sadness	1661	1526	1795
Anger	1701	1500	1902
Fear	1599	1432	1766
Disgust	1512	1364	1661
Neutral	1720	1569	1871

Note: Across the whole sample: (a) for emotion recognition, reaction times for the following emotions differed significantly from each other: happiness vs. sadness, happiness vs. fear, anger vs. fear. (b) Significant differences in perspective taking across the whole sample were evident in faster reaction times for happiness than for all other emotions, as well as for neutral vs. anger and neutral vs. fear. (c) For affective responsiveness, reaction times for happiness were significantly faster than for all other emotions except disgust, and reaction times for disgust were faster than for anger, sadness, and neutral (across the whole sample).
